# Assessing the Epidemic Potential of RNA and DNA Viruses

**DOI:** 10.3201/eid2212.160123

**Published:** 2016-12

**Authors:** Mark E.J. Woolhouse, Liam Brierley, Chris McCaffery, Sam Lycett

**Affiliations:** University of Edinburgh, Edinburgh, UK

**Keywords:** emerging viruses, epidemic potential, RNA viruses, DNA viruses, viruses, outbreaks, transmission, zoonoses

## Abstract

Many new and emerging RNA and DNA viruses are zoonotic or have zoonotic origins in an animal reservoir that is usually mammalian and sometimes avian. Not all zoonotic viruses are transmissible (directly or by an arthropod vector) between human hosts. Virus genome sequence data provide the best evidence of transmission. Of human transmissible virus, 37 species have so far been restricted to self-limiting outbreaks. These viruses are priorities for surveillance because relatively minor changes in their epidemiologies can potentially lead to major changes in the threat they pose to public health. On the basis of comparisons across all recognized human viruses, we consider the characteristics of these priority viruses and assess the likelihood that they will further emerge in human populations. We also assess the likelihood that a virus that can infect humans but is not capable of transmission (directly or by a vector) between human hosts can acquire that capability.

## Abstract 2

Detecting and quantifying transmission is a challenge needed for assessing the public health threat of emerging viruses.

A series of recent emerging infectious disease outbreaks, including the 2014 Ebola virus disease (EVD) epidemic in West Africa and the continuing Zika virus disease epidemic in the Americas, have underlined the need for better understanding of which kinds of pathogens are most likely to emerge and cause disease in human populations. Many, although not all, emerging infectious diseases are caused by viruses, and these frequently emerge from nonhuman host reservoirs (*1**–**3*). The enormous diversity (*4*) and high rates of evolution (*5*) of viral pathogens discourage attempts to predict with any precision which ones are most likely to emerge in humans. However, there is some consensus, at least in general terms, regarding the kinds of traits that are most essential in determining the capacity of a virus to infect, cause disease, and spread within human populations (Table 1). We focus on one of these traits, the capacity of a virus to spread from one human to another (by any transmission route other than deliberate laboratory exposure), a key determinant of the epidemic potential of a virus.

**Table 1:** Virus traits potentially relevant for capacity to emerge and cause disease in human populations*

Trait	Definition
Reservoir host relatedness	Viruses derived from specific host taxa (e.g., other primate species might be of increased concern)
Virus relatedness	Particular virus taxa might be predisposed to infect, cause disease, and transmit among humans
Virus host range	Viruses with a broad or narrow host range might be of greatest concern
Evolvability	Higher substitution rates might make it easier for some viruses to adapt to human hosts
Host restriction factors	Host factors, many still to be identified, are a barrier to viral infection and help determine which viruses can and cannot emerge
Transmission route	Certain transmission routes might predispose viruses to emerge in humans
Virulence	Certain virus or host factors might determine whether a virus causes mild or severe disease in humans
Host−virus coevolution	Lack of a shared evolutionary history might be associated with higher virulence

*Adapted from Morse et al. (*3*).

A theoretical framework for studying the dynamics of infectious disease outbreaks is well established (*6*). The capacity of an infectious disease to spread in a host population can be quantified in terms of its basic reproduction number, *R*_0_. *R*_0_ is defined as the average number of secondary cases generated by a single primary case in a large, previously unexposed host population, and its value tells us a great deal about the epidemiology of a pathogen. *R*_0_ = 0 indicates no spread in that population; this value would apply to zoonotic infections that do not spread between humans. *R*_0_ in the range 0<*R*_0_<1 indicates that chains of transmission are possible but that outbreaks will ultimately be self-limiting. *R*_0_>1 indicates that major epidemics can occur or that the disease may become endemic in that host population. A higher value of *R*_0_ also indicates that a greater reduction in transmission rates must be achieved to control an epidemic (*6*). *R*_0_ values have been estimated for >60 common human pathogens (*7*), including human influenza A virus (*R*_0_<2), measles virus (*R*_0_<18), and dengue virus (*R*_0_<22).

*R*_0_ is determined by a combination of pathogen traits, such as its transmission biology, which is itself a complex interplay between the within-host dynamics of the pathogen and the host response to infection, and host traits, such as demography, behavior, genetics, and adaptive immunity. Consequently, for any given infectious disease, *R*_0_ can vary between host species and between host populations. Infectious diseases with *R*_0_ close to 1 are a particular concern because small changes in their epidemiologies can lead to major changes in the threat they pose to public health (*8*).

*R*_0_ is closely related to another conceptual approach to disease emergence, the pathogen pyramid. There are different versions of this scheme (*3*,*9*). We consider a pyramid of 4 levels (Figure 1). Level 1 represents the background chatter of pathogens to which humans are continually or sporadically exposed but most of which are not capable of causing infection. Other levels can be considered in terms of the *R*_0_ of the pathogen in humans: level 2 corresponds to *R*_0_ = 0, level 3 to 0<*R*_0_<1, and level 4 to *R*_0_>1.

**Figure 1:**
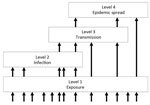
Pathogen pyramid for RNA and DNA viruses. Level 1 indicates viruses to which humans are exposed but which do not infect humans. Level 2 indicates viruses that can infect humans but are not transmitted from humans. Level 3 indicates viruses that can infect and be transmitted from humans but are restricted to self-limiting outbreaks. Level 4 indicates viruses that are capable of epidemic spread in human populations. Transitions between levels (indicated by arrows) correspond to different stages of virus emergence in human populations. Reprinted from Woolhouse et al. (*10*).

## Data and Analysis

### Identifying and Characterizing Level 3 and 4 Viruses

We updated our previous systematic literature review (*10*) of the capacity of virus species to transmit between humans (i.e., level 3 and level 4 viruses; Technical Appendix). Such viruses are found in 25 of 29 families containing viruses that infect mammals or birds (discounting 2 reports of family *Nodaviridae* species in mammals/birds). The 4 exceptions comprise 2 families that have no known human-infective viruses (*Arteriviridae* and *Birnaviridae*) and 2 with species that have been reported in humans but only at level 2 (*Asfarviridae* and *Bornaviridae*).

A total of 22 of these families contain level 4 viruses with epidemic potential in humans (sometimes described as human-adapted viruses) (*11*). This finding indicates that this capability is widely distributed among virus taxa. The 3 families with level 3 viruses but no level 4 viruses are the *Arenaviridae*, *Bunyaviridae*, and *Rhabdoviridae*.

A list of 37 presumptive level 3 virus species is provided in Table 2. These species cover a wide taxonomic range and a variety of transmission routes, including vectorborne. Several level 3 viruses have historically been associated with sizeable outbreaks (>100 cases) in human populations: Bwamba, Oropouche, Lake Victoria Marburg, Sudan Ebola, and o’nyong-nyong viruses. For some other viruses, including Guanarito, Junin, lymphocytic choriomeningitis, and Sabia (all arenaviruses); simian virus 40; Titi monkey virus; and influenza A(H5N1) virus, human-to-human transmission is rare or merely suspected. In addition, several viruses are only known or believed to transmit between humans by iatrogenic routes or vertical transmission; this group (Table 2) might be regarded as unlikely epidemic threats.

**Table 2:** Viruses (n = 37) that are known or suspected of being transmissible (directly or indirectly) between humans but to date have been restricted to short transmission chains or self-limiting outbreaks*

Genome, virus family	Virus name
Single-stranded RNA (ambisense)	
Arenaviruses	Guanarito, Junin, Lassa, Lujo, Machupo, Sabia, Dandenong,* lymphocytic choriomeningitis*
Bunyaviruses	Andes, Bwamba, Crimean-Congo hemorrhagic fever, Oropouche, Rift Valley, severe fever with thrombocytopenia syndrome
Single-stranded RNA (positive sense)	
Flaviviruses	Japanese encephalitis,* Usutu,* West Nile*
Coronaviruses	Middle East respiratory syndrome
Togaviruses	Barmah Forest, o’nyong-nyong, Ross River, Semliki Forest, Venezuelan equine encephalitis
Single-stranded RNA (negative sense)	
Filoviruses	Bundibugyo Ebola, Lake Victoria Marburg, Sudan Ebola
Paramyxoviruses	Nipah
Rhabdoviruses	Bas-Congo, rabies*
Double-stranded RNA	
Reoviruses	Nelson Bay, Colorado tick fever*
Double-stranded DNA	
Adenoviruses	Titi monkey
Herpesviruses	Macacine herpesvirus 1
Polyomaviruses	Simian virus 40
Poxviruses	Monkeypox, Orf, vaccinia

*Human transmission of these viruses is known only by iatrogenic or vertical routes.

When a virus is transmitted by a vector, it can be particularly difficult to confirm or exclude the infectiousness of human cases, as with Semliki forest, Barmah forest, and Rift Valley fever viruses. Similarly, even when human–vector–human transmission is believed to occur, it is often difficult to quantify its contribution to a given outbreak, as with Venezuelan equine encephalitis virus.

Level 2 viruses are those that can infect humans (>100 species) but have never been reported to be transmitted by humans (*10*). In at least some instances, such as influenza A(H5N1) virus (*12*), this finding is attributable to tissue tropisms during human infection that are incompatible with onward transmission.

Shifts in pyramid level equate to shifts in the public health threat posed by a virus. We consider possible shifts in the following sections.

### Level 1 to Levels 3 and 4

Virus species of mammalian and, more rarely, avian origin are sometimes observed to be transmissible between humans when first found in humans, which constitutes a jump from level 1 straight to level 3 or 4 (Figure 1), and events of this kind have been reported regularly. Recent examples that appear on the basis of available evidence to fit this model include severe acute respiratory syndrome coronavirus (first reported in humans in 2003), Bundibugyo Ebolavirus (2008), Lujo virus (2009), severe fever with thrombocytopenia syndrome virus (2011), and Middle East respiratory syndrome coronavirus (MERS-CoV) (2012).

We still have incomplete knowledge of the diversity of viruses that infect mammals and birds; the few hundred recognized species (*4*) surely represent only a small fraction of the total (*3*). Moreover, we have few predictors of potential human-to-human transmissibility. One possible indicator is emergence from nonhuman primates, with suggestions that primate viruses are more likely to be able to, or to acquire the ability to, spread in human populations (*13**,**14*). However, emergence of human transmissible viruses from bat (e.g., severe acute respiratory syndrome coronavirus) or bird (e.g., influenza) reservoirs indicates that this trait is associated with a wide range of reservoirs.

### Level 2 to Levels 3 and 4

The possibility that level 2 viruses might acquire the capacity to be transmitted between humans (i.e., move into level 3 or 4) is a major concern, especially in the context of influenza A(H5N1) virus and other avian influenza virus subtypes. However, there are few examples of this transition throughout the entire recorded history of human viruses going back to 1901. One possible example involves the simian immunodeficiency virus (SIV) and HIV. A SIV_smm_-derived laboratory strain of SIV has been reported to infect humans, but without onward transmission (*15*). SIV_smm_ is related to HIV-2. SIV_cpz_, which is related to HIV-1, has not been directly observed in humans. However, different HIV-1 lineages, independently derived from SIV_cpz_, are variably transmissible in humans, and the pandemic HIV-1 M lineage was the only virus to overcome a key host restriction factor (human tetherin) (*16*). The only other examples of viruses newly transmitted between humans relate to rare instances of iatrogenic transmission (e.g., Colorado tick fever or rabies viruses).

Epidemiologic and phylogenetic considerations routinely inform our assessment of the likelihood of human-to-human transmission being observed in the future. For example, there is markedly less concern about rabies virus than about avian influenza virus, and we suggest 2 reasons for this observation. First, rabies virus has a much longer history of and a much higher incidence of human infection, but human-to-human transmission is extremely rare. Second, there is no evidence that other rhabdoviruses viruses (with the possible exception of Bas-Congo virus, which represents a novel genus) are transmissible in humans (or primates more generally).

### Level 3 to Level 4

Level 3 viruses can also become level 4 viruses. We note that virus evolution is not (necessarily) required for *R*_0_ to become >1 in human populations. Differences in host (or vector) behavior, ecology, or demography might be sufficient (*8*).

Instances of shifts from level 3 to level 4 in recent times have been infrequent. Three candidates are Ebolavirus, Zika virus, and chikungunya virus. However, although these viruses have caused epidemics of unprecedented size in humans populations in the past decade, the condition *R*_0_>1 in human populations might had been previously met for all 3 viruses (*17**–**19*).

For Ebola virus, the epidemic in West Africa in 2014 constituted the first appearance of this virus in high-density, urban populations, which is expected to correspond to a higher value of *R*_0_. The chikungunya virus epidemic in the Indian Ocean region in 2005 was associated with a vector species jump (from *Aedes aegypti* to *Ae. albopictus* mosquitoes) that has been linked to a mutation in the virus envelope 1 protein gene (*18*). The chikungunya virus epidemic in the Caribbean region in 2013 followed the first appearance of chikungunya in the Americas and infected populations that had no history of exposure to the virus. The current Zika virus epidemic in South America appears to be another example of a transition from a level 3 to a level 4 arbovirus associated with geographic spread into areas with high densities of vectors (*19*). Occasional Zika virus transmission directly from infected humans to other humans are of considerable interest, but probably contribute little to *R*_0_.

Chikungunya, Zika and the other level 4 arboviruses (yellow fever and dengue viruses) illustrate that, for arboviruses, a high potential for spread in human populations is linked to carriage by anthropophilic vector species, particularly mosquitoes of the genus *Aedes*. In contrast, no tick species are regarded as anthropophilic, and there are no level 4 and few level 3 tickborne arboviruses.

### Epidemiologic Patterns

The preceding sections illustrate that identifying transitions of viruses between level 2 and level 3 or between level 3 and level 4 is not always straightforward. Standard epidemiologic theory can help clarify our expectations.

**Figure 2:**
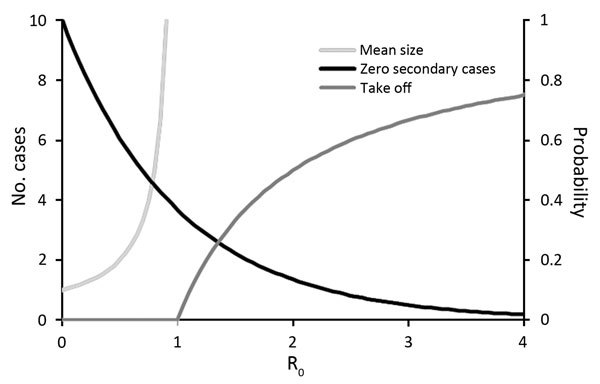
Expected outbreak dynamics for RNA and DNA viruses given a single primary case in a large, previously unexposed host population, as a function of the basic reproduction number *R*_0_. Mean size of outbreak as total number of cases (*N*) is given by N = 1/(1 − *R*_0_) for *R*_0_<1 (light gray line, left axis). Probability of 0 secondary cases (i.e., outbreak size N = 1) is given by *P*_1_ = exp(−*R*_0_) (black line, right axis). Probability of a major outbreak is given by *P*_takeoff_ = 1 – 1/*R*_0_ for *R*_0_>1 (dark gray line, right axis).

As we discussed, pyramid level is related to the basic reproduction number *R*_0_ in human populations. In turn, the value of *R*_0_ is indicative of expected outbreak dynamics. Some key results (Figure 2) are the probability that a single primary case will generate >1 secondary cases (for any value of *R*_0_), the expected average size of an outbreak generated (over the range 0<*R*_0_<1), and the probability that an epidemic will spread in the human population (for *R*_0_>1). These results strictly apply to homogeneous infections in a homogeneous host population, although more general frameworks can accommodate host or pathogen heterogeneity (*20**–**22*). Nonetheless, the key predictions that secondary cases do not always occur even if *R*_0_>0 and that major epidemics do not always occur even if *R*_0_>1 are robust.

**Figure 3:**
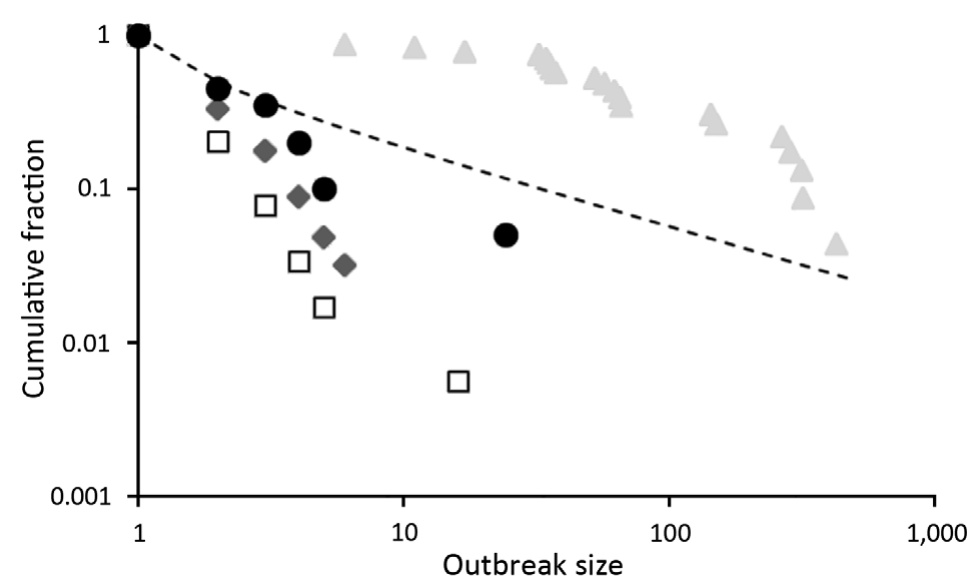
Distribution of outbreak sizes for RNA and DNA viruses as plots of outbreak size *x* (horizontal axis) versus fraction of outbreaks of size >*x* (vertical axis), both on logarithmic scales. Data are shown for 4 infectious diseases. Squares indicates Andes virus disease in South America (*24*); diamonds indicate monkeypox in Africa (*26*); circles indicate Middle East respiratory syndrome in the Middle East (*25*); and triangles indicate filovirus (all species) diseases in Africa before 2013 (*27*). For comparison, expected values for the case *R*_0_ = 1, obtained from the expression for the probability of an outbreak of size >*x*, *P*(*x*) = Γ(*x* – 1/2)/√πΓ(*x*), are also shown (dashed line). Data for filoviruses are not consistent with expectation for *R*_0_<1.

From an epidemiologic perspective, our confidence that a putatively level 2 virus is truly incapable of human-to-human transmission is thus a function of the number of index cases observed. The transition between level 3 and level 4 can be studied in terms of the expected distribution of outbreak sizes (*23*). In the range 0<*R*_0_<1, an overdispersed distribution of outbreak sizes is expected: most outbreaks are small (often just single cases) with a long tail of larger outbreaks. This pattern has been reported for a range of emerging viral diseases (Figure 3). As the critical threshold *R*_0_ = 1 is approached, this value is signaled in the outbreak size distribution (Figure 3). This framework has been used successfully to monitor the epidemiology of measles virus in the United Kingdom after a decrease in childhood vaccination rates in the late 1990s and indicated the approach to the critical threshold that corresponded to loss of herd immunity (*23*).

Outbreak size distribution analysis has been applied to human case data for Andes virus (*24*), monkeypox virus (*20*), and MERS-CoV (*25*) (Figure 3). For EVD up to 2013, data are clearly inconsistent with theoretical expectation for *R*_0_ <1 (Figure 3), which suggests that large numbers of small outbreaks have remained undetected or that *R*_0_ was already >1 in at least some settings. Either way, *R*_0_ ≈1 for EVD in humans implies that small differences in the biology or epidemiology of the virus would lead to large changes in scale of outbreaks (*8*), which could make events such as the EVD epidemic in 2014, if not predictable, then much less unexpected.

### Evolution

Changes in pyramid level might be mediated by virus evolution or changes in virus ecology (*28*). A major issue is whether the capacity of a virus to spread in human populations arises as a result of adaptation (evolution of transmissibility that occurs during human infection) or preadaptation (genetic variation within nonhuman reservoirs that predisposes a virus not only to infect humans but also transmit between humans, noting that RNA viruses often show high levels of genetic variation such that they are sometimes described as quasi-species [*29*]). These alternatives have been characterized as tailor-made and off-the-shelf, respectively (*28*). The first alternative implies a progression from no or low transmissibility between humans to moderate or high transmissibility. The second alternative implies moderate or high transmissibility at first infection of humans.

We consider that our survey of documented changes of pyramid level is most consistent with the off-the-shelf model of virus emergence. In particular, we can find no convincing examples of level 2 viruses becoming level 3 or 4 viruses, which suggests that, if this happens at all, it typically happens sufficiently rapidly (i.e., requires a sufficiently small number of introductions) that we fail to observe the level 2 phase. In contrast, we regularly observe viruses at levels 3 or 4 the first time they are detected in human populations.

Nonetheless, the possibility of virus evolution of transmissibility in a new host has been demonstrated experimentally for influenza A(H5N1) virus in ferrets (*30*). A theoretical study (*31*) suggested that the fact that this virus subtype has been circulating widely in poultry populations, with frequent human exposure and sporadic human infection for almost 20 years, provides little or no reassurance about its future evolutionary trajectory.

HIV lineages show clear evidence of adaptation to humans (*16*), but as discussed earlier, it is not clear whether the SIV lineages that gave rise to HIV-1 or HIV-2 were capable of transmission between human hosts. We speculate that extended infection times make tailor-made emergence more likely for retroviruses.

### Transmission

Demonstrating that an infected human has the potential to transmit the infection to another human is not always straightforward. High virus titers in body secretions and excretions, blood, or skin are considered indicative. Case clusters are suggestive, but if persons occupy the same environment (e.g., household), then it might be difficult to rule out common exposure. Case clusters must be epidemiologically plausible (i.e., delimited in space and time in a manner consistent with the known or assumed epidemiology of the virus). Genotyping techniques are useful tools for confirming a cluster but do not resolve the source of infection.

For several of the viruses we studied (e.g., Bas-Congo, Lujo, Nelson Bay, and severe fever with thrombocytopenia syndrome viruses) (Table 2), the evidence for human-to-human transmission is best regarded as tentative, particularly where putative clusters were small. Such assessments can be even more difficult for vectorborne viruses. In many situations, the best evidence for the human-to-human transmission will come from analysis of virus genome sequences.

### Phylogenetic Analysis

One approach to resolving the question of human-to-human transmission is analysis of nucleotide sequence data, sometimes referred to as forensic phylogenetics. Nucleotide substitution rates in fast-evolving RNA viruses, such as MERS-CoV and Ebola virus, are ≈1–5 × 10^−3^/site/year (*32**,**33*), making it possible to use sequences isolated from different hosts at different times to estimate time-resolved phylogenetic trees. Estimates of the transmission chain from temporal sequence data can be improved by incorporating additional information on the date of onset of individual cases, duration of latent and infectious periods, and overall prevalence (*34*).

**Figure 4:**
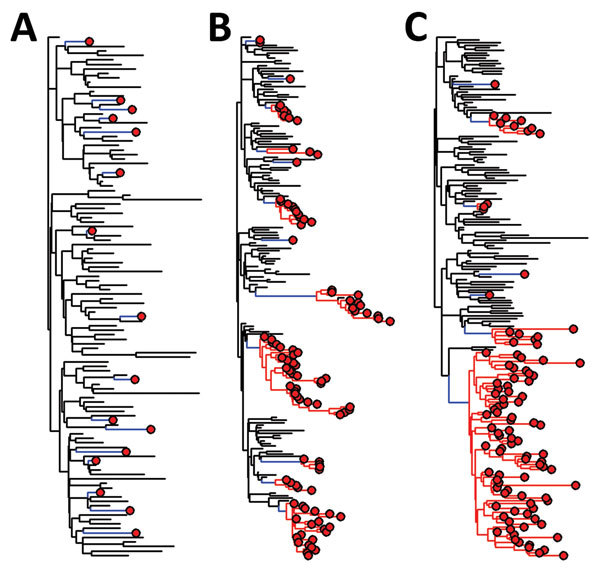
Phylogenetic trees for simulated emerging infectious disease outbreaks caused by RNA and DNA viruses in a mixed population of 1,000 human and 5,000 nonhuman hosts. Trees were constructed by using a standard susceptible–infected–removed model (*6*). For each of 3 infection scenarios in nonhuman hosts (black lines), rare zoonotic transmission events (blue lines), human-to-human transmission (red lines), and human cases (red circles) are indicated. For the nonhuman population *R*_0_ = 2 throughout. Transmissibility within the human populations varies from A) spillover: no human−human transmission (*R*_0_ = 0); B) limited human−human transmission with *R*_0_ = 1; and C) epidemic spread within humans (*R*_0_>1). A maximum of 100 infections are randomly sampled from each population in each simulated outbreak.

We provide some example phylogenetic trees generated from simulated epidemics (Figure 4). In an epidemic in an animal reservoir with occasional transmission to humans (Figure 4, panel A), for each human sequence, the most closely related next sequence is of animal origin. Clusters of closely related human sequences are shown, and the distribution of the expected cluster sizes is a function of *R*_0_ (Figure 4, panels B, C) (*35*).

In an outbreak, it might be difficult to find and sample the putative source animal cases. However, estimating the time to most recent common ancestor (TMRCA) of the human cases will indicate how long the infection has been spreading. For sporadic zoonoses (Figure 4, panel A), most transmission has occurred unobserved in the animal reservoir, and the TMRCA of pairs of human cases will be long because these sequences are not closely related. For outbreaks involving human-to-human transmission (Figure 4, panels B, C), the TMRCA of the cluster of human cases will be closer to the date of the first human infection (whether sampled or not) and provides the estimated date of the zoonotic event.

Use of sequence data to distinguish between multiple instances of human infection from a common animal source and human-to-human transmission in the early stages of an outbreak is extremely challenging because of short timescales, and involvement of few mutations. However, genetic differences and phylogenetic evidence show that at least 2 of the first 3 reported cases of influenza A (H7N9) virus infection in humans were believed to originate from distinct domestic avian sources (*36*).

Further sequencing of avian samples implied that a low-pathogenicity influenza A(H7N9) virus strain had been spreading in domestic birds for ≈1 year before sporadic cases were detected in humans (*37*). Similarly, detection of genetically distant lineages of MERS-CoV, which persisted for only a few months each, suggest multiple introductions from an animal reservoir and only limited human-to-human transmission to date (*32*). In contrast, the influenza A(H1N1) pandemic in 2009 and the EVD epidemic in West Africa in 2014 were believed to be the results of single zoonotic events, followed by sustained human-to-human transmission (*33*), as shown by a single rapidly expanding lineage.

## Conclusions

Our survey of the capacity of RNA and DNA virus infections to be transmitted, directly or indirectly, between humans leads to several conclusions and practical suggestions for improving surveillance of emerging infectious diseases and targeting efforts to identify future public health threats. In support of these conclusions, the World Health Organization recently published list of priority emerging infectious diseases and corresponding viruses (*38*) included 6 of the viruses in Table 2.

A major observation is that the taxonomic diversity of viruses that are possible threats to public health is wide, but bounded. Most human infective viruses are closely related to viruses of other mammals and some to viruses of birds. There are no indications that humans acquire new viruses from any other source. However, diversification within human populations occurs and is a prominent feature of some DNA virus taxa (e.g., family *Papillomaviridae*) (*4*).

In general, however, our knowledge of origins of human viruses is still incomplete. Although the origins of HIV-1 have been extensively investigated (*16*), for most other viruses, even level 4 viruses, little or no research has occurred. An origins initiative (*9*) would help establish the routes into human populations that have been used by other viruses.

Transmissibility within human populations is a key determinant of epidemic potential. Many viruses that can infect humans are not capable of being transmitted by humans; most human transmissible viruses that emerge already have that capability at first human infection or acquire it relatively rapidly. If transmission from humans would require a change in a phylogenetically conserved trait, such as tissue tropism or transmission route (*4*), then such viral paradigm shifts will probably be extremely rare (*39*).

Even when a virus is capable of transmission between humans, the critical threshold *R*_0_>1 is not always achieved. However, because changes in virus traits or host population characteristics can influence *R*_0_, level 3 viruses (Table 2) are of special interest from a public health perspective, and of special concern when, like MERS-CoV, they also cause severe illness. Demonstrating human transmissibility is often difficult, but essential. The best evidence is likely to come from virus genome sequencing studies. These studies should be a public health priority (*40*).

We currently have few clues to help us predict which mammalian or avian viruses might pose a threat to humans and, especially, which might be transmissible between humans. One argument in favor of experimental studies of these traits, including controversial gain of function experiments (*30*), is that they could help guide molecular surveillance for high-risk virus lineages in nonhuman reservoirs.

The first line of defense against emerging viruses is effective surveillance (*40*). A better understanding of which kinds of viruses in which circumstances pose the greatest risk to human health would enable evidence-based targeting of surveillance efforts, which would reduce costs and increase probable effectiveness of this endeavor.

Technical Appendix Literature search protocol used to obtain information on pyramid level for assessing the epidemic potential of RNA and DNA viruses.
