# Supplementary material for: Assessing the Epidemic Potential of RNA and DNA Viruses
Source: Emerg Infect Dis. 2016 Dec;22(12):2037–44. doi: 10.3201/eid2212.160123 (PMC5189130; doi:10.3201/eid2212.160123)
Supplement: Technical Appendix — Literature search protocol used to obtain information on pyramid level for assessing the epidemic potential of RNA and DNA viruses. [file 16-0123-Techapp-s1.pdf]

Article DOI: http://dx.doi.org/10.3201/eid2212.160123

Assessing the Epidemic Potential of RNA and DNA Viruses

Technical Appendix

Literature Search Parameters

For each virus species, we used the search terms comprising the virus name (including all synonyms sourced from King et al. [*1*] as a list separated by OR) AND human to human, OR person to person, OR interhuman, OR communicab*, OR transmi*, OR outbreak, OR cluster, OR household, OR iatrogenic, OR urban cycle. We searched Google Scholar, ProMED-mail (*2*), original references in Woolhouse et al. (*3*), and obtained R_0 estimates from Hay et al. (*4*).

References were compiled for each virus, and the virus was assigned to pyramid level independently by 2 of the authors (L.B. and C.M.). The key references providing evidence of human transmissibility for the level 3 viruses (Table 2 in the text) are in the Technical Appendix Table.

References

1. King AM, Lefkowitz E, Adams MJ, Carstens EB. Virus taxonomy: ninth report of the International Committee on Taxonomy of Viruses. Amsterdam: Elsevier; 2012.

2. Madoff LC. ProMED-mail: an early warning system for emerging diseases. Clin Infect Dis. 2004;39:227–32. PubMed http://dx.doi.org/10.1086/422003

3. Woolhouse ME, Adair K, Brierley L. RNA viruses: a case study of the biology of emerging infectious diseases. Microbiol Spectr. 2013;1:OH-0001–2012. PubMed http://dx.doi.org/10.1128/microbiolspec.OH-0001-2012

4. Hay SI, Battle KE, Pigott DM, Smith DL, Moyes CL, Bhatt S, et al. Global mapping of infectious disease. Philos Trans R Soc Lond B Biol Sci. 2013;368:20120250. PubMed http://dx.doi.org/10.1098/rstb.2012.0250

5. Palacios G, Druce J, Du L, Tran T, Birch C, Briese T, et al. A new arenavirus in a cluster of fatal transplant-associated diseases. N Engl J Med. 2008;358:991–8. PubMed http://dx.doi.org/10.1056/NEJMoa073785

6. de Manzione N, Salas RA, Paredes H, Godoy O, Rojas L, Araoz F, et al. Venezuelan hemorrhagic fever: clinical and epidemiological studies of 165 cases. Clin Infect Dis. 1998;26:308–13. PubMed http://dx.doi.org/10.1086/516299

7. Rugiero HR, Parodi AS, Gotta H, Boxaca M, Olivari AJ, Gonzalez E. Epidemic hemorrhagic fever. Laboratory infection and inter-human passage [in Spanish]. Rev Asoc Med Argent. 1962;76:413–7. PubMed

8. Frame JD, Baldwin JM Jr, Gocke DJ, Troup JM. Lassa fever, a new virus disease of man from West Africa. I. Clinical description and pathological findings. Am J Trop Med Hyg. 1970;19:670–6. PubMed

9. Briese T, Paweska JT, McMullan LK, Hutchison SK, Street C, Palacios G, et al. Genetic detection and characterization of Lujo virus, a new hemorrhagic fever-associated arenavirus from southern Africa. PLoS Pathog. 2009;5:e1000455. PubMed http://dx.doi.org/10.1371/journal.ppat.1000455

10. Fischer SA, Graham MB, Kuehnert MJ, Kotton CN, Srinivasan A, Marty FM, et al.; LCMV in Transplant Recipients Investigation Team. Transmission of lymphocytic choriomeningitis virus by organ transplantation. N Engl J Med. 2006;354:2235–49. PubMed http://dx.doi.org/10.1056/NEJMoa053240

11. Peters CJ, Kuehne RW, Mercado RR, Le Bow RH, Spertzel RO, Webb PA. Hemorrhagic fever in Cochabamba, Bolivia, 1971. Am J Epidemiol. 1974;99:425–33. PubMed

12. Peters CJ, Jahrling PB, Khan AS. Patients infected with high-hazard viruses: scientific basis for infection control. In: Schwarz DT, Siegl PD, editors. Imported virus infections. Vienna: Springer; 1996. p. 141–68.

13. Padula PJ, Edelstein A, Miguel SD, López NM, Rossi CM, Rabinovich RD. Hantavirus pulmonary syndrome outbreak in Argentina: molecular evidence for person-to-person transmission of Andes virus. Virology. 1998;241:323–30. PubMed http://dx.doi.org/10.1006/viro.1997.8976

14. Tomori O, Monath TP, Lee V, Fagbami A, Fabiyi A. Bwamba virus infection: a sero-survey of veterbrates in five ecological zones in Nigeria. Trans R Soc Trop Med Hyg. 1974;68:461–5. PubMed http://dx.doi.org/10.1016/0035-9203(74)90069-8

15. Burney MI, Ghafoor A, Saleen M, Webb PA, Casals J. Nosocomial outbreak of viral hemorrhagic fever caused by Crimean hemorrhagic fever-Congo virus in Pakistan, January 1976. Am J Trop Med Hyg. 1980;29:941–7. PubMed

16. Pinheiro FP, Travassos da Rosa AP, Travassos da Rosa JF, Ishak R, Freitas RB, Gomes ML, et al. Oropouche virus. I. A review of clinical, epidemiological, and ecological findings. Am J Trop Med Hyg. 1981;30:149–60. PubMed

17. Go YY, Balasuriya UB, Lee CK. Zoonotic encephalitides caused by arboviruses: transmission and epidemiology of alphaviruses and flaviviruses. Clin Exp Vaccine Res. 2014;3:58–77. PubMed http://dx.doi.org/10.7774/cevr.2014.3.1.58

18. Gai Z, Liang M, Zhang Y, Zhang S, Jin C, Wang S-W, et al. Person-to-person transmission of severe fever with thrombocytopenia syndrome bunyavirus through blood contact. Clin Infect Dis. 2012;54:249–52. PubMed http://dx.doi.org/10.1093/cid/cir776

19. Memish ZA, Zumla AI, Al-Hakeem RF, Al-Rabeeah AA, Stephens GM. Family cluster of Middle East respiratory syndrome coronavirus infections. N Engl J Med. 2013;368:2487–94. PubMed http://dx.doi.org/10.1056/NEJMoa1303729

20. Wamala JF, Lukwago L, Malimbo M, Nguku P, Yoti Z, Musenero M, et al. Ebola hemorrhagic fever associated with novel virus strain, Uganda, 2007–2008. Emerg Infect Dis. 2010;16:1087–92. PubMed http://dx.doi.org/10.3201/eid1607.091525

21. Hennessen W. Epidemiology of Marburg virus disease. In: Martini G, Siegert R, editors. Marburg virus disease. Berlin: Springer; 1971. p. 161–5.

22. World Health Organization. Ebola haemorrhagic fever in Sudan, 1976. Bull World Health Organ. 1978;56:247–70. PubMed

23. Fischer M, Lindsey N, Staples JE, Hills S; Centers for Disease Control and Prevention (CDC). Japanese encephalitis vaccines: recommendations of the Advisory Committee on Immunization Practices (ACIP). MMWR Recomm Rep. 2010;59:1–27. PubMed

24. Cavrini F, Gaibani P, Longo G, Pierro AM, Rossini G, Bonilauri P, et al. Usutu virus infection in a patient who underwent orthotropic liver transplantation, Italy, August–September 2009. Euro Surveill. 2009;14:19448. PubMed

25. Centers for Disease Control and Prevention. West Nile virus infection in organ donor and transplant recipients—Georgia and Florida, 2002. MMWR Morb Mortal Wkly Rep. 2002;51:790. PubMed

26. Hsu VP, Hossain MJ, Parashar UD, Ali MM, Ksiazek TG, Kuzmin I, et al. Nipah virus encephalitis reemergence, Bangladesh. Emerg Infect Dis. 2004;10:2082–7. PubMed http://dx.doi.org/10.3201/eid1012.040701

27. Randall WH, Simmons J, Casper EA, Philip RN. Transmission of Colorado tick fever virus by blood transfusion—Montana. MMWR Morb Mortal Wkly Rep. 1975;24:422, 427.

28. Chua KB, Crameri G, Hyatt A, Yu M, Tompang MR, Rosli J, et al. A previously unknown reovirus of bat origin is associated with an acute respiratory disease in humans. Proc Natl Acad Sci U S A. 2007;104:11424–9. PubMed http://dx.doi.org/10.1073/pnas.0701372104

29. Grard G, Fair JN, Lee D, Slikas E, Steffen I, Muyembe J-J, et al. A novel rhabdovirus associated with acute hemorrhagic fever in central Africa. PLoS Pathog. 2012;8:e1002924. PubMed http://dx.doi.org/10.1371/journal.ppat.1002924

30. Houff SA, Burton RC, Wilson RW, Henson TE, London WT, Baer GM, et al. Human-to-human transmission of rabies virus by corneal transplant. N Engl J Med. 1979;300:603–4. PubMed http://dx.doi.org/10.1056/NEJM197903153001105

31. Lindsay M, Johansen C, Broom AK, Smith DW, Mackenzie JS. Emergence of Barmah Forest virus in Western Australia. Emerg Infect Dis. 1995;1:22–6. PubMed http://dx.doi.org/10.3201/eid0101.950104

32. Mathiot CC, Grimaud G, Garry P, Bouquety JC, Mada A, Daguisy AM, et al. An outbreak of human Semliki Forest virus infections in Central African Republic. Am J Trop Med Hyg. 1990;42:386–93. PubMed

33. Rosen L, Gubler DJ, Bennett PH. Epidemic polyarthritis (Ross River) virus infection in the Cook Islands. Am J Trop Med Hyg. 1981;30:1294–302. PubMed

34. Weaver SC, Salas R, Rico-Hesse R, Ludwig GV, Oberste MS, Boshell J, et al.; VEE Study Group. Re-emergence of epidemic Venezuelan equine encephalomyelitis in South America. Lancet. 1996;348:436–40. PubMed http://dx.doi.org/10.1016/S0140-6736(96)02275-1

35. Chen EC, Yagi S, Kelly KR, Mendoza SP, Tarara RP, Canfield DR, et al. Cross-species transmission of a novel adenovirus associated with a fulminant pneumonia outbreak in a new world monkey colony. PLoS Pathog. 2011;7:e1002155. PubMed http://dx.doi.org/10.1371/journal.ppat.1002155

36. Centers for Disease Control. Epidemiologic notes and reports. B-virus infection in humans—Pensacola, Florida. MMWR Morb Mortal Wkly Rep. 1987;36:289–90, 295–6. PubMed

37. Barbanti-Brodano G, Martini F, Corallini A, Lazzarin L, Trabanelli C, Vignocchi B, et al. Reactivation of infectious simian virus 40 from normal human tissues. J Neurovirol. 2004;10:199–205. PubMed http://dx.doi.org/10.1080/13550280490441112

38. Breman JG, Kalisa-Ruti, Steniowski MV, Zanotto E, Gromyko AI, Arita I. Human monkeypox, 1970-79. Bull World Health Organ. 1980;58:165–82. PubMed

39. Nagington J, Whittle CH. Human orf. Isolation of the virus by tissue culture. BMJ. 1961;2:1324–7. PubMed http://dx.doi.org/10.1136/bmj.2.5263.1324

40. Zafar A, Swanepoel R, Hewson R, Nizam M, Ahmed A, Husain A, et al. Nosocomial buffalopoxvirus infection, Karachi, Pakistan. Emerg Infect Dis. 2007;13:902–4. PubMed http://dx.doi.org/10.3201/eid1306.061068

Technical Appendix Table. References providing evidence of human transmission of 38 level 3 viruses in Table 2 of the text

Virus	Primary reference
Dandenong virus	(*5*)
Guanarito virus	(*6*)
Junin virus	(*7*)
Lassa virus	(*8*)
Lujo virus	(*9*)
Lymphocytic choriomeningitis virus	(*10*)
Machupo virus	(*11*)
Sabia virus	(*12*)
Andes virus	(*13*)
Bwamba virus	(*14*)
Crimean-Congo hemorrhagic fever virus	(*15*)
Oropouche virus	(*16*)
Rift Valley fever virus	(*17*)
Severe fever with thrombocytopenia syndrome virus	(*18*)
Middle East respiratory syndrome coronavirus	(*19*)
Bundibugyo Ebolavirus	(*20*)
Lake Victoria Marburgvirus	(*21*)
Sudan Ebolavirus	(*22*)
Japanese encephalitis virus	(*23*)
Usutu virus	(*24*)
West Nile virus	(*25*)
Nipah virus	(*26*)
Colorado tick fever virus	(*27*)
Nelson Bay orthoreovirus	(*28*)
Bas-Congo virus	(*29*)
Rabies virus	(*30*)
Barmah Forest virus	(*31*)
O’nyong-nyong virus	(*32*)
Ross River virus	(*33*)
Semliki Forest virus	(*32*)
Venezuelan equine encephalitis virus	(*34*)
Titi monkey adenovirus	(*35*)
Macacine herpesvirus 1	(*36*)
Simian virus 40	(*37*)
Monkeypox virus	(*38*)
Orf virus	(*39*)
Vaccinia virus	(*40*)
